# Medical Ethics

**Published:** 1843-05-27

**Authors:** 


THE MEDICAL EXAMINER.

PHILADELPHIA, MAY 27, 1843.

### MEDICAL ETHICS.

We understand the College of Physicians of Philadelphia has been occupied recently, at several of its meetings, revising the by-laws which govern its fellows. Such a revision and emendation are perhaps necessary—we say perhaps, because we are by no means certain that corporate bodies add anything to their dignity or influence by devising rules or laws which they have no power to enforce upon those to whom they may be applicable. It is the opinion of some very respectable persons, that one of the chief defects in the operation of our political system is, its superabundant, hasty, and imperfect legislation. If the College of Physicians of Philadelphia were vested with power by the State legislature, to regulate the practice of medicine and surgery throughout the State of Pennsylvania, or even in the county of Philadelphia, it might enact salutary and effectual rules, beneficial to the members of the medical profession, and still more advantageous to the people of Pennsylvania. How far the creation of rules, which have merely the force of the opinion or declaration of the college, may influence the profession generally, must depend upon the sound, common-sense principles they embody.

The rules proposed for adoption by the college have been printed; and among them is the following:

"24. [XI.] A regular collegiate medical education furnishes the presumptive evidence of professional ability; and is so honourable and beneficial, that it gives a just claim to preëminence among physicians, in proportion to the degree in which it has been enjoyed and improved. Yet, as it *is not indispensably necessary* to the attainment of knowledge, skill, and experience, they *who have really* acquired, in a competent measure, such qualifications, without its advantages, should not be fastidiously excluded from the privileges of fellowship."

What is the substance of this declaration, and what is to be its tendency?

First; it asserts that it is highly respectable to have the degree of Doctor in Medicine conferred on one by a college or university, and that this diploma or certificate of "a regular collegiate medical education furnishes the *presumptive* evidence of professional ability."

Second; it declares that "knowledge, skill, and experience" in medicine may be attained without "a regular collegiate medical education;" consequently, those persons who have *really* become *knowing*, *skilful*, and *experienced* practitioners, without "a regular collegiate medical education," should not be considered inferior to those who have gained a diploma, and "should not be fastidiously excluded from the privileges of fellowship" of the College of Physicians?

If a "regular collegiate medical education," vouched for by a collegiate diploma, be only "presumptive" evidence of professional ability, what is the presumptive or positive evidence of professional ability to the people, or members of the College of Physicians, in practitioners who have no regular medical education, but who have "picked up" their knowledge hap-hazard or intuitively, or who are doctors by virtue of the seventh male descent. In a word, by what test, or by what means, is the college or any one of its members to discriminate between self-styled doctors and legally commissioned practitioners? How is it to be determined "who have *really* acquired, in a competent measure," the qualifications—"knowledge, skill, and experience"—without a regular medical education, that shall entitle them, on any ground, to "the privileges of fellowship" with the whole medical profession? Are they to be formally or otherwise examined by the college, or by a member of the college? or, is the question of competency to rest upon common rumor—the voice of the people? If the latter testimony be taken as presumptive of "professional ability," unless they should "be fastidiously excluded from the privileges of fellowship," homœopathists, Thomsonians, mesmerists, the St. John Longs of the day, and the venders and advocates of "red drop," and Devere's specific, may be all, one day, numbered among the fellows of the College of Physicians? They should not be "fastidiously" excluded because they keep a professional card in a newspaper, merely stating the locality of their office, or because their names may be attached to a popular remedy? What is the virtual difference in the merit or demerit attached to the individuals whose names designate a remedy or an instrument? We once heard this expression,—"I mean Wistar, the lozenge man!" and although it sounded gratingly upon our ears, it occurred to us that the public, especially where the individuals are not known, must regard "Wistar, the lozenge man," Sherman, the lozenge man, Wright, Brandreth, Morrison and Lee, the pill men and panacea men, as equally entitled to its consideration and confidence? What is the difference between Sir Astley Cooper and Dr. Hull in this respect? Why should it be, in the eyes of any College of Physicians or Medical Society, honourable to Sir Astley Cooper to have a knife designated by his name, and at the same time disreputable for another surgeon to have a truss known by his name? Because the inventor of the truss derives peculiar advantage from it through a patent, while the inventor of the knife gives it to the whole profession, without tax or hindrance of any kind. But the same inventor of the knife thought himself entitled to peculiar advantages for his lectures and teachings, and consequently laid an injunction upon the publishers of what is commonly known as the "pirated edition" of Sir Astley Cooper's lectures. Why shall a man be allowed the advantages of patent right for a book he may write, but be disgraced among his professional brothers, if he claim a share of the profits arising from an extensively useful instrument of his own invention? And will the college "fastidiously" exclude from the privileges of fellowship those who have "the presumptive evidence of professional ability" in "a regular collegiate medical education," because they hold a patent for an instrument of their own invention, and take in as fellows the irregularly instructed practitioner who has invented nothing!

The tendency of this article of the proposed by-law is to discredit universities and colleges, and regular medical education. While we admit that knowledge and skill may be acquired without resorting to the halls of a college, we believe that instances of the kind alluded to are rare; for this reason it would be much better to exclude from the privileges of fellowship with the profession, all those who do not possess formal and legitimate evidence that they have acquired a "competent" knowledge of the science and principles of the healing art. This admission that a regular medical collegiate education "is not indispensably necessary to the attainment of knowledge," is calculated to lower the standard of information which has been already brought too low by the competition of the medical schools—a competition in reference to the number, and not the competency of the graduates—and the consequence is, that the public is rapidly losing confidence in the value of medical diplomas; a higher though more uncertain test is required—popular favour; and for the service of the army and navy, scrutinizing examinations, which are much more likely to be authentic tests of professional qualification.

Before we can determine what description of practitioners should be admitted to the privileges of fellowship, it would be well to define, as nearly as may be, what qualifications and attributes constitute a physician, and then what testimony shall be considered as sufficient to satisfy us that the aspirant is worthy of the title. There should be some high standard set up that cannot be overturned by pecuniary considerations—and perhaps, under the present system of instruction, no college that is supported by fees from students has wealth and influence enough to require very high qualifications for its diploma. But the College of Physicians of Philadelphia, or any similar institution in the United States, might create a standard that it would be highly honourable to be successfully measured by. There must be a line drawn somewhere, to separate the irregular practitioners and empirics from the regularly educated physicians, or in a very few years the public will not distinguish learned and high-toned physicians from advertising doctors:—the latter persons may say, it is true I never received medical instruction at any college, but you know, knowledge, skill and experience, can be as well acquired out of a college as in it; and I had no money to throw away upon professors: besides, I am as capable of treating disease, and living by my practice, as the most learned and fastidious among them.

With proper deference to the joint wisdom of the college, we are of opinion that the article 24 is neither judicious nor politic, in its present form.

There is another article (33) which ought to be entirely omitted, or given in an opposite sense. It is as follows:

"33. [XVIII.] Ministers of the gospel should be attended gratuitously by the faculty. This is the acknowledged rule; but such of the clergy as are qualified, either from their incomes or fortunes, to make a reasonable remuneration for medical attendance, are not to be more exempted than any other order of patients."

According to our experience, there is no class or calling, taken as a body, better able to pay physician's bills, and no class of men more ungrateful for medical attendance than the clergy. And what is equally worthy of remark, there is no class of men who do more to sustain medical quackery than ministers of the gospel. We have known a clergyman to introduce a professed electro-magnetist to a patient who was, to the clergyman's knowledge, receiving the professional attentions of a respectable practitioner, and countenance a proposal that electro-magnetism should be resorted to without the knowledge of the physician!

Another clergyman — a popular divine — circulated pamphlets among his sick parishioners, lauding the infallible efficacy of brandy and salt. Some advocate homœopathy and its kindred delusions, and others squint in favour of surgical quackery. While we feel pity for this exhibition of mental weakness, and find a disposition to pardon it on the plea of morbid benevolence, we do not think that the simple fact of a man being a minister of the gospel entitles him to the gratuitous services of any physician for himself or family.

The services of the physician or surgeon are as valuable as those of any other man in the community, and we can perceive no reason why any profession or calling should be more entitled to them than another. Poor lawyers are as much entitled, in our opinion, to gratuitous advice, as poor clergymen;—in short, sickness and poverty, no matter upon what calling they may come together, are claims upon the time and attention that no true physician will pass unheeded. But we are opposed to declaring that clergymen ought to be attended gratuitously by the faculty—for, as a general rule, the favour is not so much to the clergyman, perhaps, as to the congregation over which he presides. And when we see a church making collections to defray the expenses of a European tour for its pastor, it seems to us the same means might pay the demands of the physician. Why should the physician belonging to a congregation be more taxed than any other member of it? He is expected to pay his church dues with the rest, but there is no reason why he should labour as the family physician of the pastorage.

According to our views, it is not the way to sustain the profession, to cut off from its members any legitimate sources of profit. To the great mass of its members the profession is the source of their living—of their bread; and if the course of professional charity is prescribed in this, or in any other way, the actual value of the profession to its members is reduced. The high tone that should grace every medical practitioner must yield, sooner or later, to poverty; and we know of instances where this cause alone has driven respectable and regularly educated physicians to advertise in the newspapers, and, consequently, cut loose from the ethics of a profession that did not give them bread. And what may be regretted by some, their course secured them and their families against starvation.

We think that all kinds of medical certificates, whether for life insurance offices, beneficent associations, exemption from military service, or for pensions, are legitimate objects of profit; but the practitioner should be left to decide for himself the particular cases for charging, and the amount of the fee.* And, in all cases, the cer-

* We have heard of a medical certificate that procured for a man, who was in the annual receipt of $3000, a sum of $10,000 in hand, and $300 a year besides. Yet the physician got no fee.

tificate given by a physician should be studiously frank and conscientiously correct, to such an extent that he would not hesitate to make oath that it contained his real and undisguised opinion.

According to the proposed rule, (34.) "These testimonials, unless under particular circumstances, should be considered as acts due to the public, and, therefore, not to be compensated by any gratuity."

May it not be respectfully asked, through what process, or catenation of circumstances, have physicians become debtors to the public, in such wise as to permit this same public to claim their professional services whenever they may be requisite? Does the public set up any claim of this nature over the services of other professional men? Are legal opinions ever required by the public, or by individuals, without acknowledging the right of a fee, at the hands of any persons connected with the administration of the laws, judges, jurors, lawyers, bailiffs or witnesses? Their time and their knowledge are paid for at established rates; and why should a physician be told:—Sir, you must state your opinion to the court as to the health of a juryman—it is a duty you owe the public, and consequently you are not entitled to remuneration? Or we may even suppose a stronger case. The public requires your opinion, whether an individual died of disease or of violence. Is this opinion of value to the public? Yes; but it cannot be "compensated by any gratuity." Why? The opinion of the judges and jurors is compensated; the public pays the prosecuting attorney, the witnesses, and every body except the physician!

Again: Is the opinion of a physician valuable in cases of life insurance? Certainly. To whom is medical opinion valuable? To the Life Insurance Company! Then, on what principle of equity or justice has that company obtained a right over what is valuable, in form of service—a sort of pilotage—without remuneration? Is it due the public that physicians should labour gratuitously to save the money of Life Insurance Companies?

